# Supplementary figures and images for: DALIA- a comprehensive resource of Disease Alleles in Arab population
Source: PLoS One. 2021 Jan 13;16(1):e0244567. doi: 10.1371/journal.pone.0244567 (PMC7806169; doi:10.1371/journal.pone.0244567)

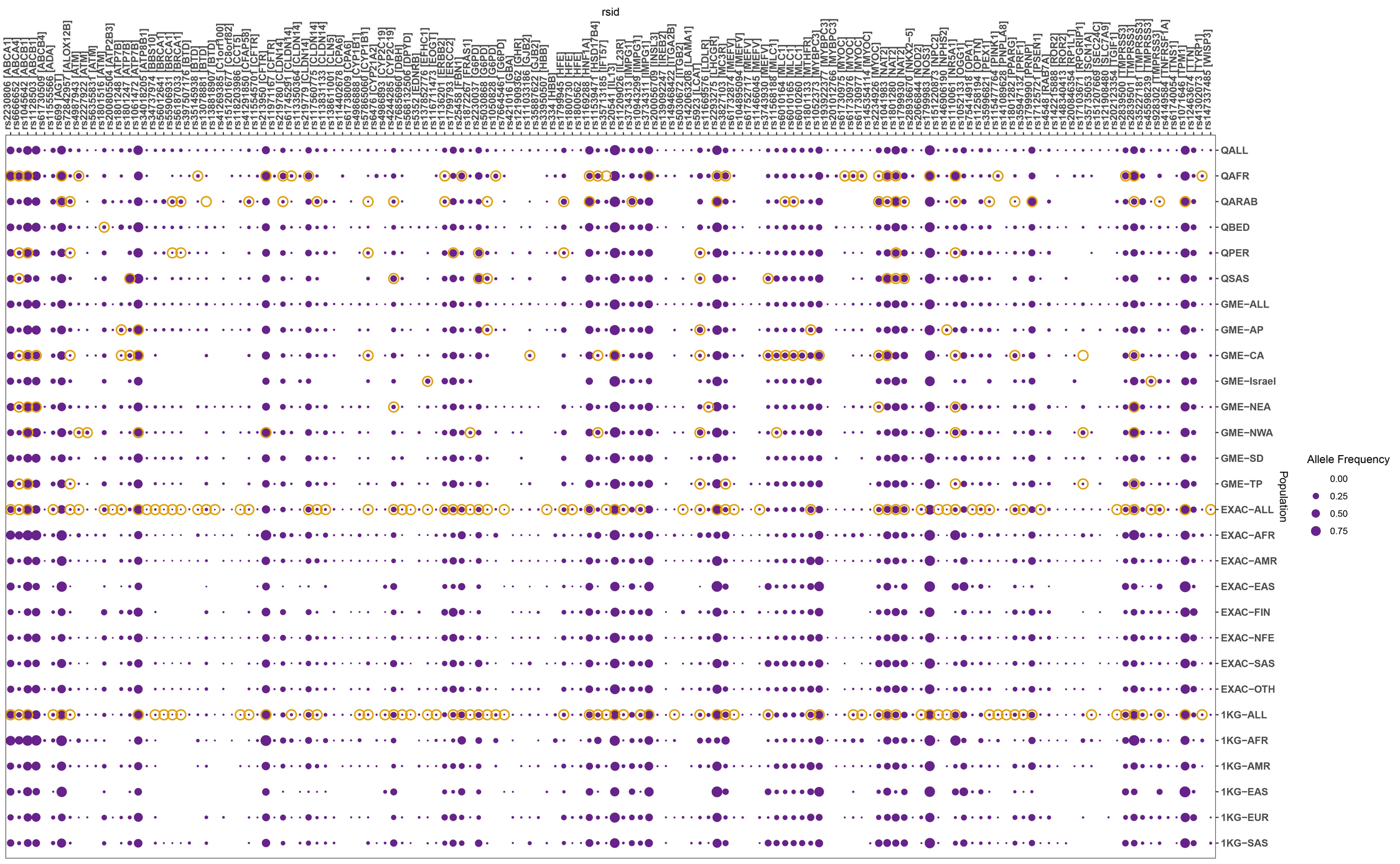

Supplement: S1 Fig — (TIF) [file pone.0244567.s001.tif]

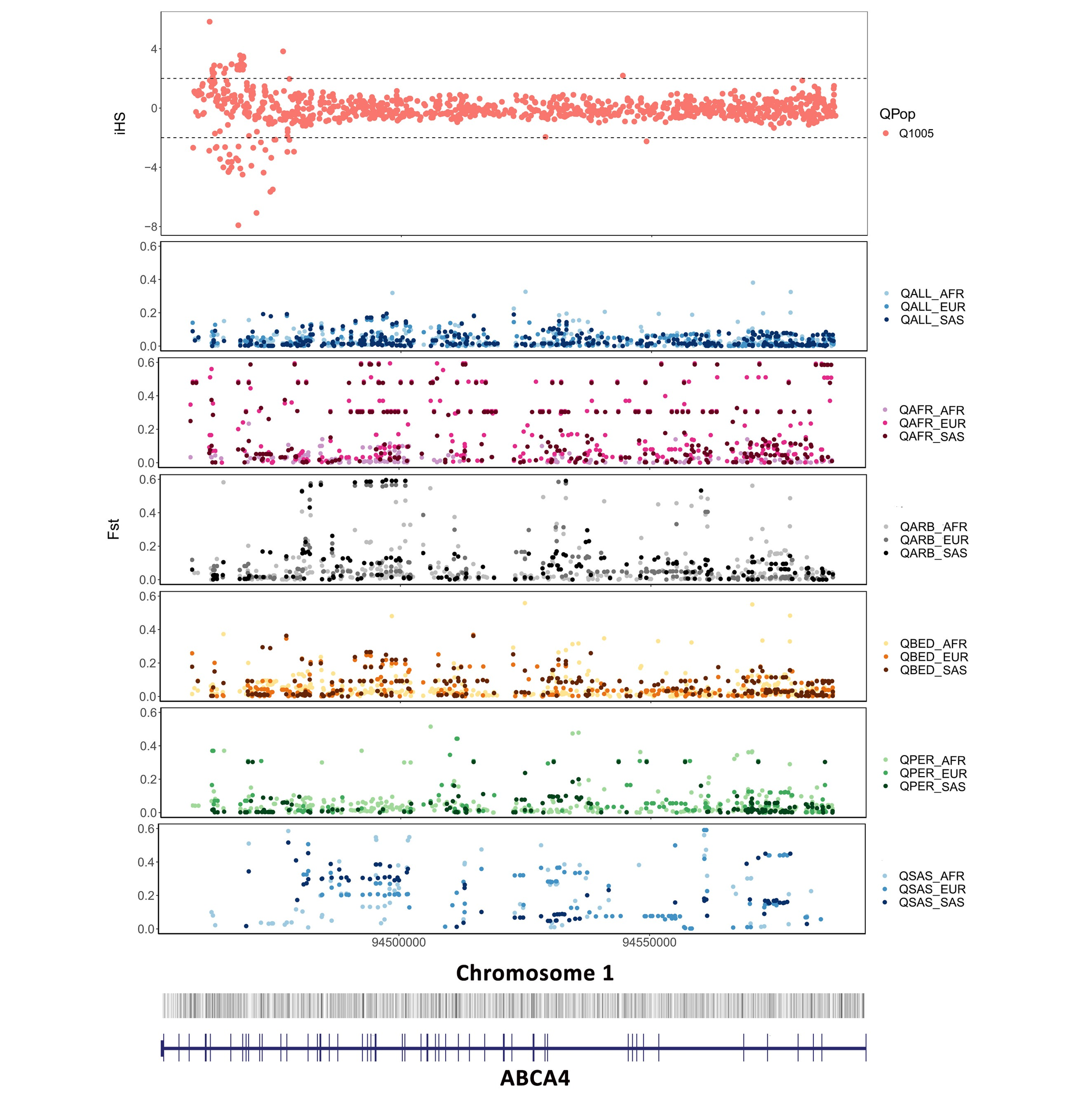

Supplement: S2 Fig — The 10 genes with top 1% |iHS| scores include ABCA4, THSD1, ATP7B, MEFV, ADA, CLDN14, MLC1, HSD17B4, NAT2, and ABCA1 (S1 Table). This plot depicts the iHS as well as pairwise Fst scores along the gene loci, for all the known variants for the gene ABCA4. The array of lines at the bottom represent all known variants, and the exon structure of the gene is shown beneath it. (TIF) [file pone.0244567.s002.tif]

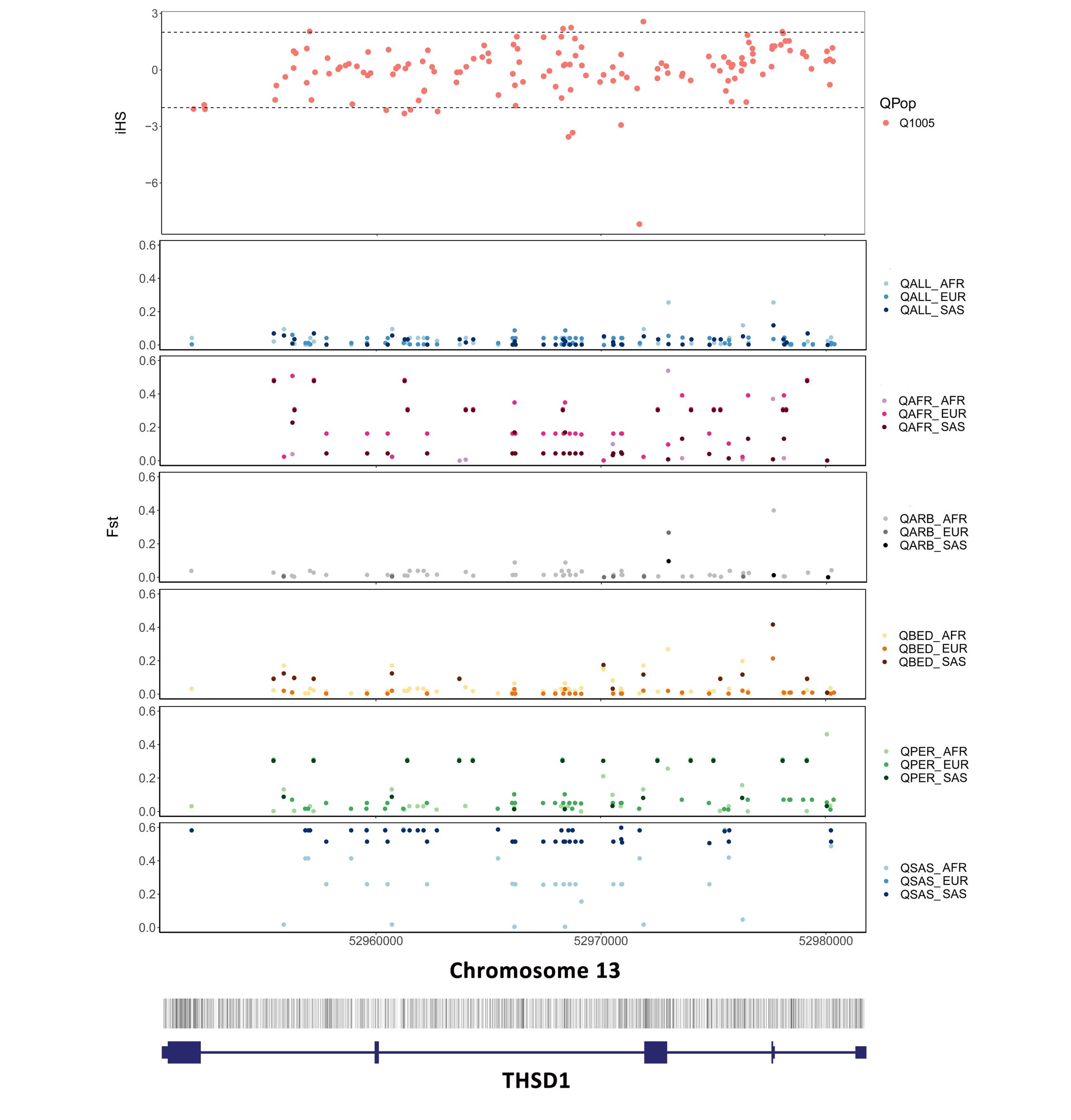

Supplement: S3 Fig — (TIF) [file pone.0244567.s003.tif]

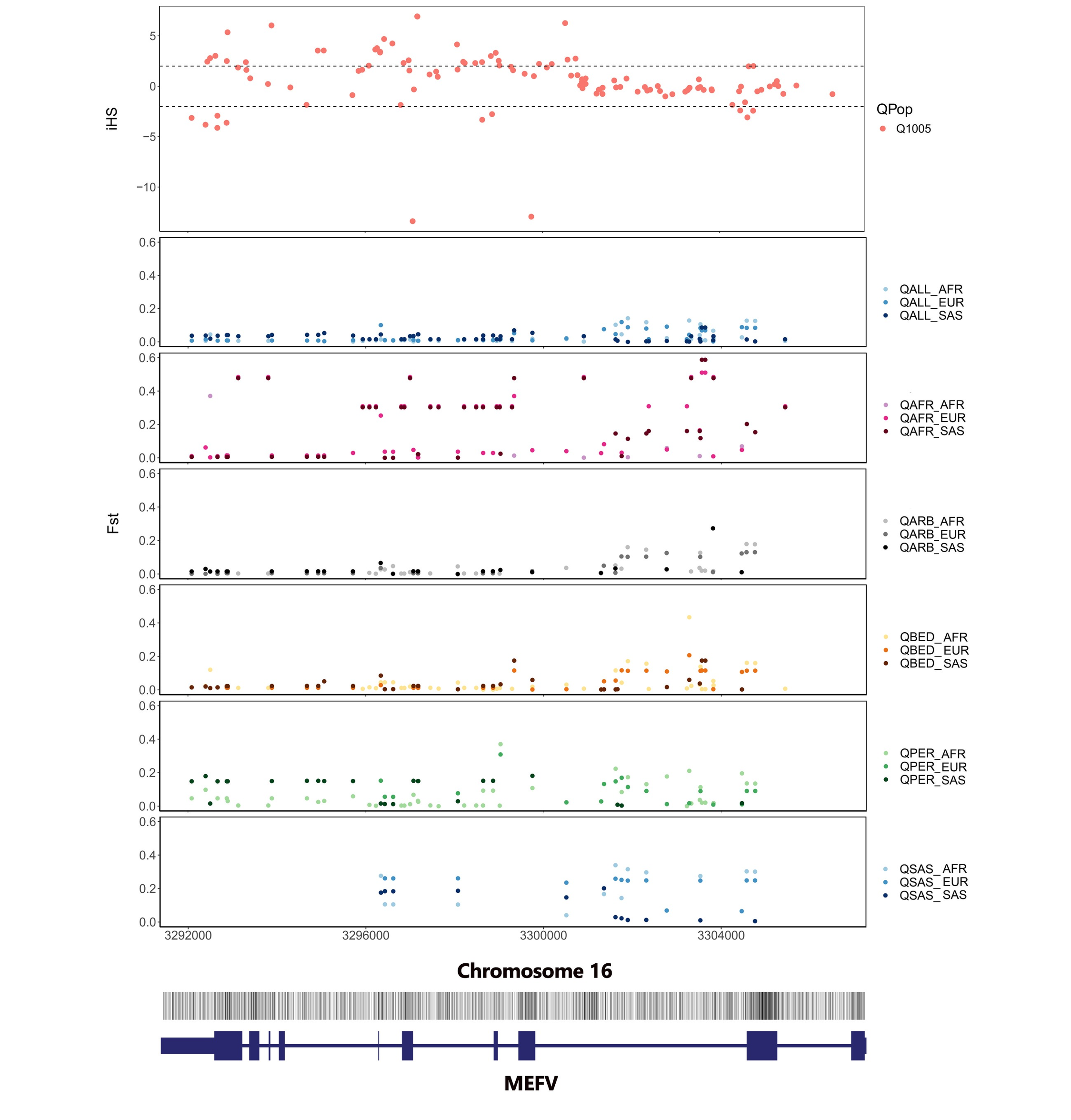

Supplement: S4 Fig — (TIF) [file pone.0244567.s004.tif]

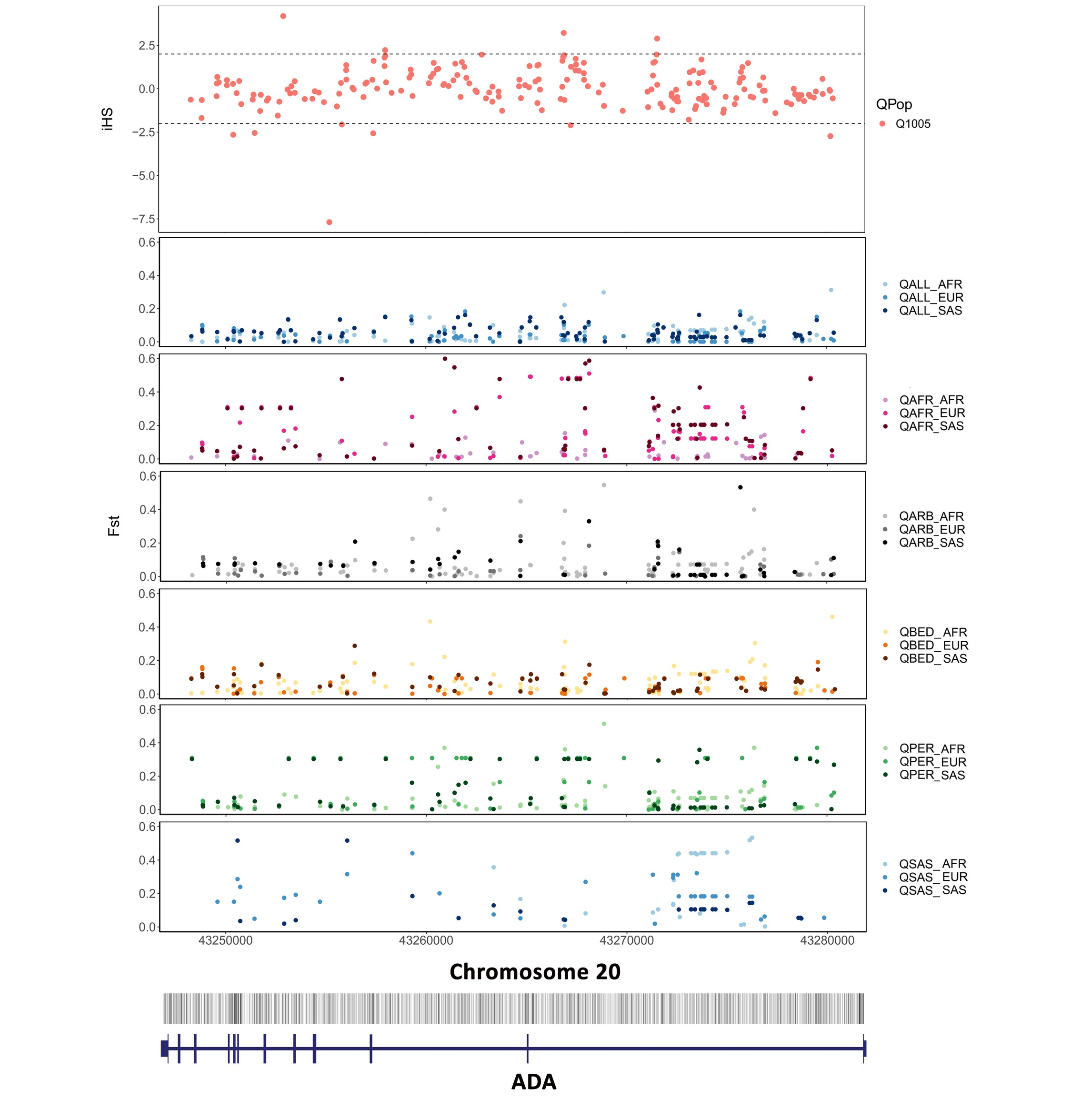

Supplement: S5 Fig — (TIF) [file pone.0244567.s005.tif]

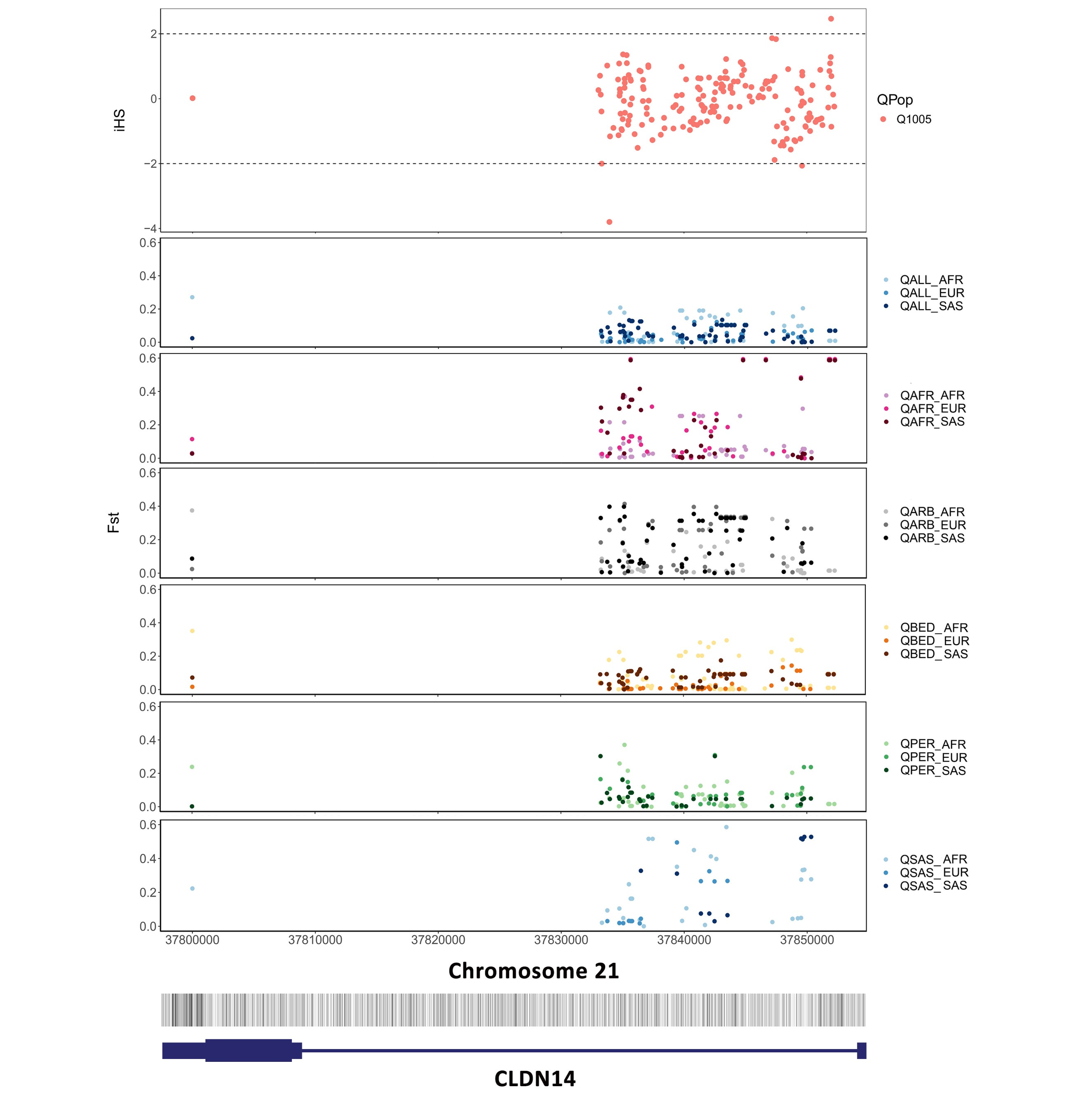

Supplement: S6 Fig — (TIF) [file pone.0244567.s006.tif]

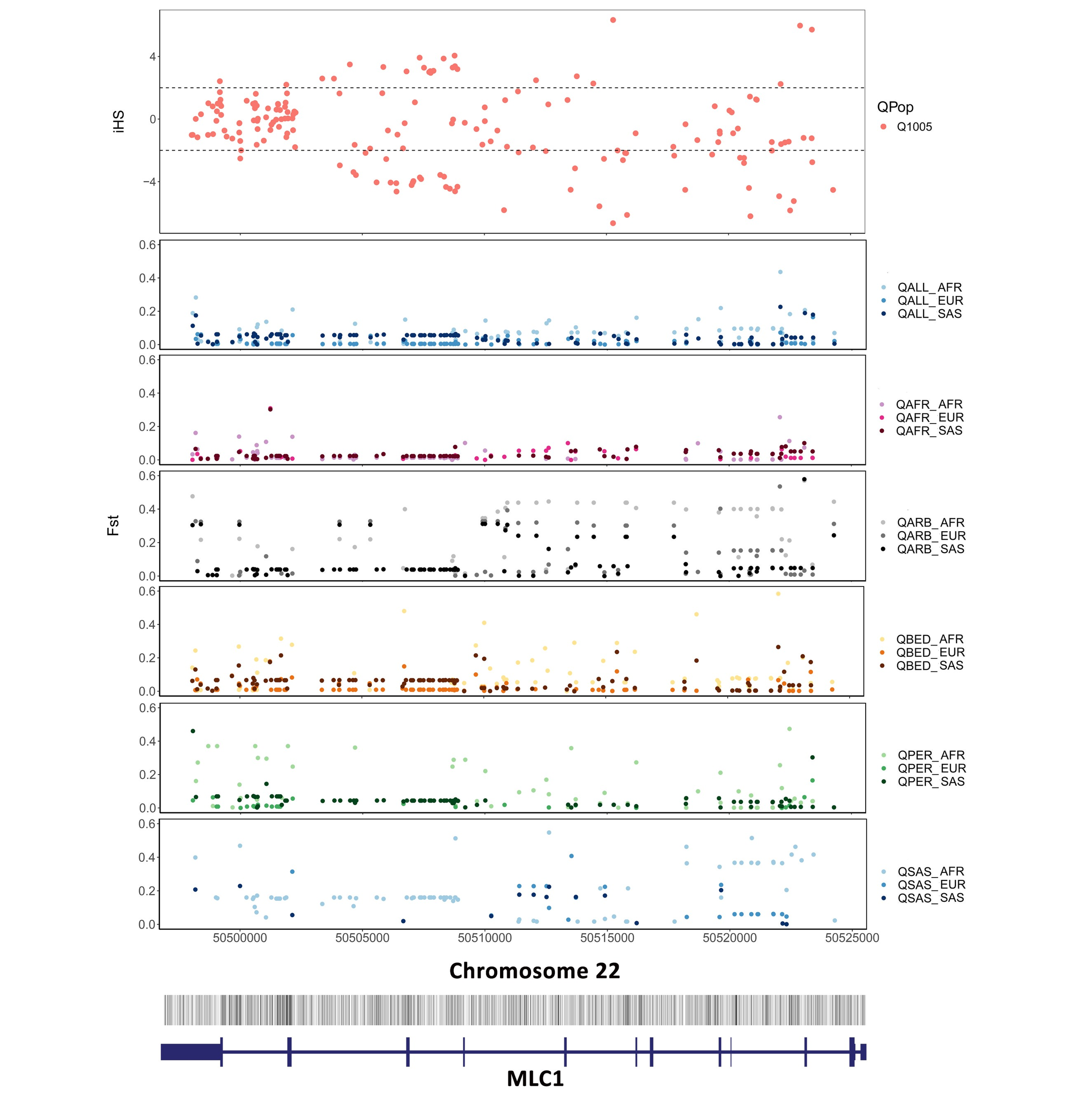

Supplement: S7 Fig — (TIF) [file pone.0244567.s007.tif]

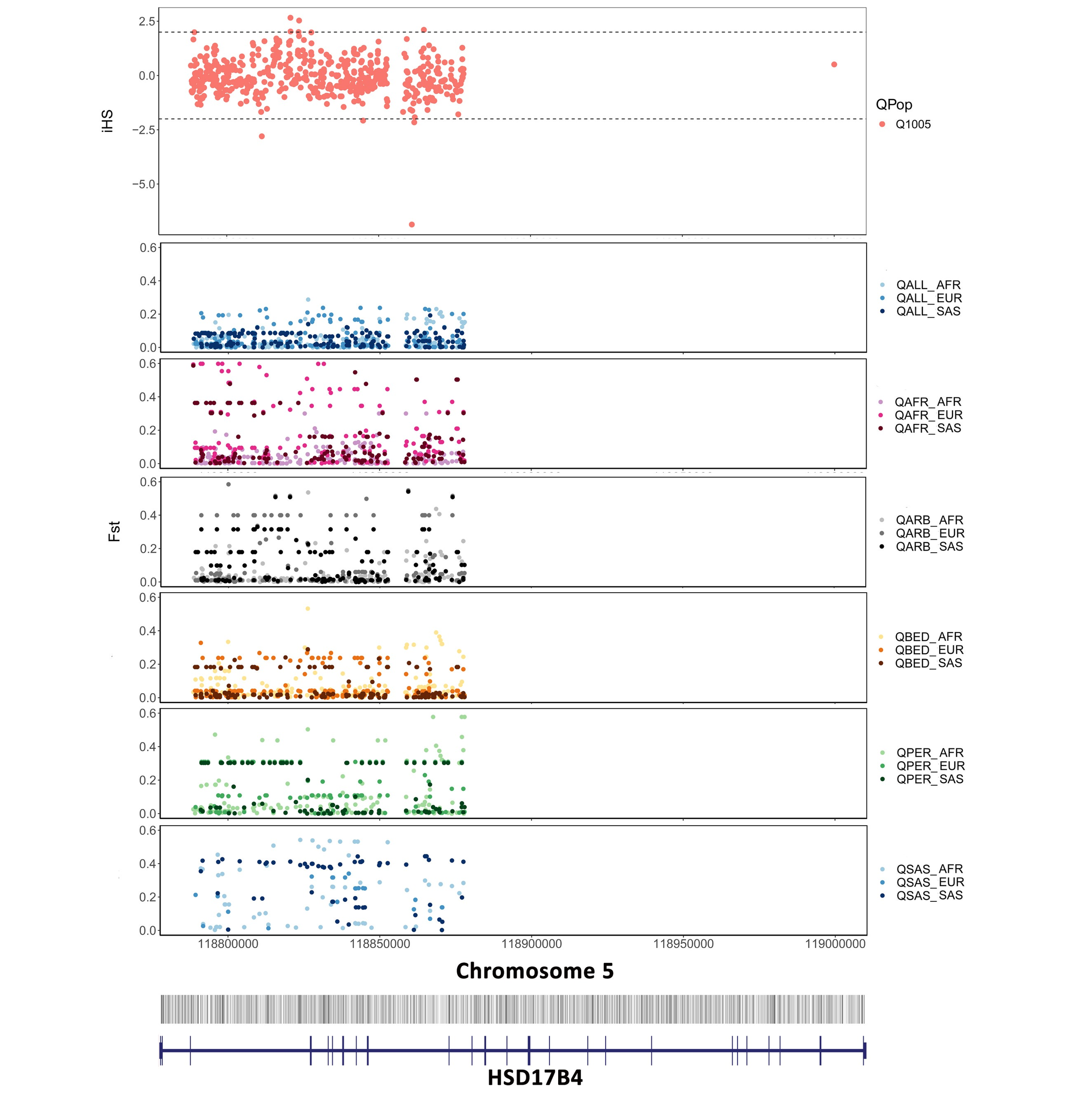

Supplement: S8 Fig — (TIF) [file pone.0244567.s008.tif]

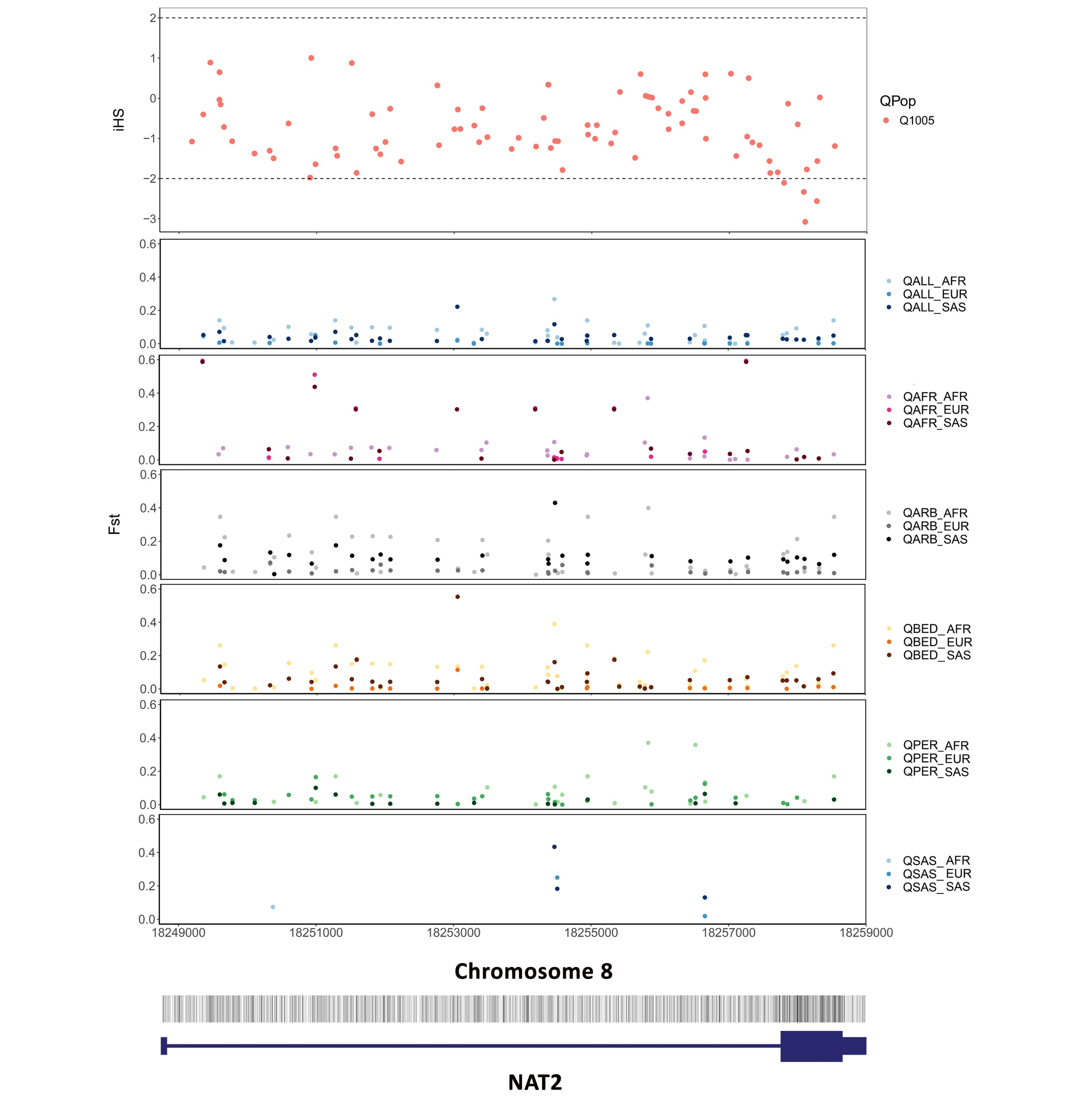

Supplement: S9 Fig — (TIF) [file pone.0244567.s009.tif]

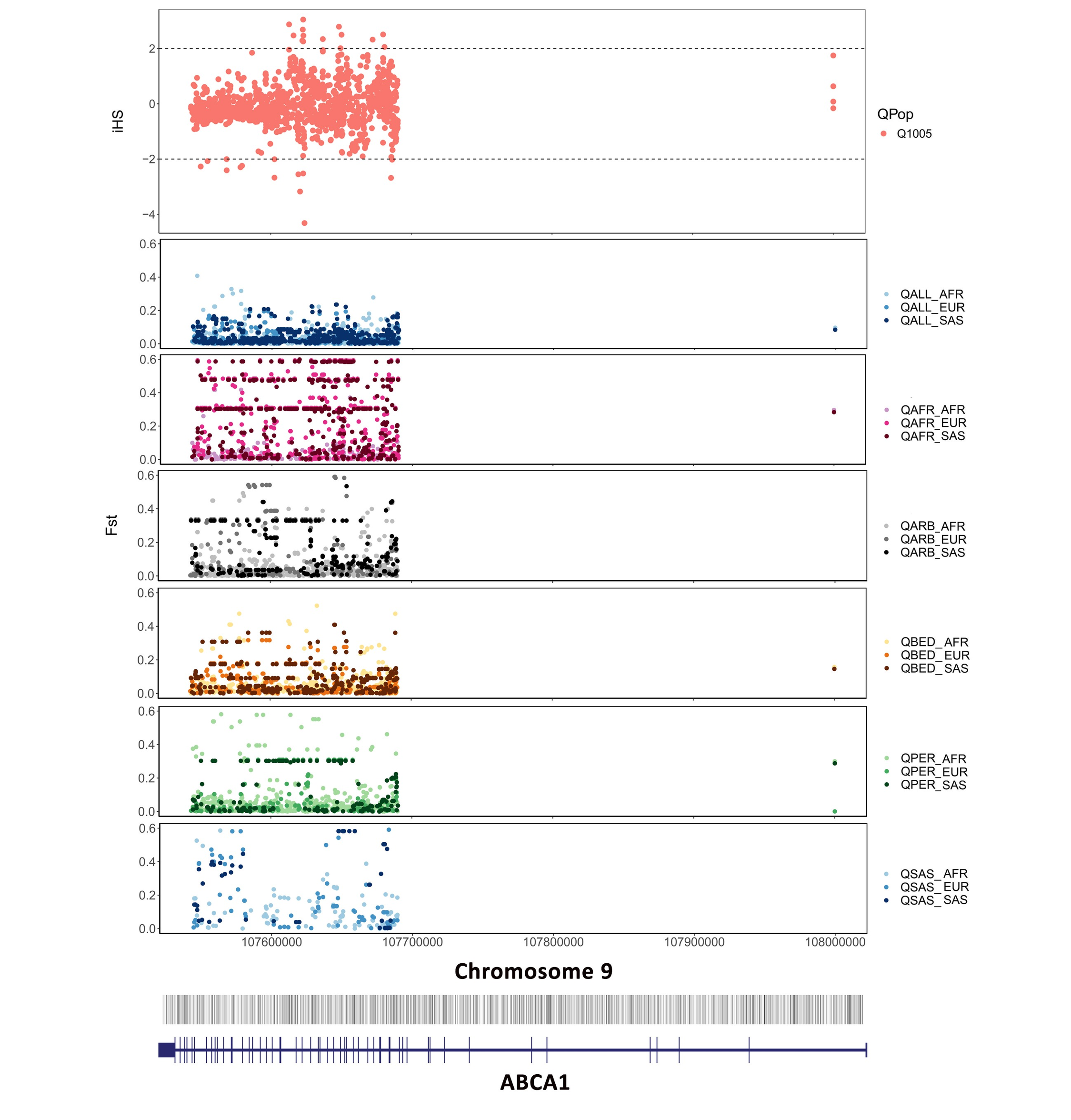

Supplement: S10 Fig — (TIF) [file pone.0244567.s010.tif]
